# 痰液中生物活性物质在肺癌诊断中的作用和意义

**DOI:** 10.3779/j.issn.1009-3419.2021.102.46

**Published:** 2021-12-20

**Authors:** 愈茗 金, 子轩 陈, 权 陈, 雷皓 沙, 诚 沈

**Affiliations:** 1 610041 成都，四川大学华西临床医学院 West China School of Medicine, Sichuan University, Chengdu 610041, China; 2 610041 成都，四川大学华西医院胸外科 Department of Thoracic Surgery, West China Hospital, Sichuan University, Chengdu 610041, China

**Keywords:** 痰液, 肺肿瘤, 生物标志物, 诊断, Sputum, Lung neoplasms, Biomarkers, Diagnosis

## Abstract

肺癌是我国目前发病率最高的恶性肿瘤之一，其诊断的金标准需要进行组织活检的病理学检查或脱落细胞学检查，二者的有创性和敏感性限制了他们的使用。痰液中含有大量核酸、蛋白质，是肺功能的良好反映物，肺癌组织也会影响痰液中的生物成分，检测其中的生物活性物质可有助于肺癌的诊断。本文综合目前国内外的研究结果，对痰液中可用于肺癌诊断的生物活性物质做一综述。

## Abstract (en)

The incidence rate of lung cancer is one of the highest incidence of malignancies in China. The gold standard for diagnosis requires pathological examination or cytological examination of biopsy. The invasive and sensitive nature of the two limits their use. Sputum contains a large number of nucleic acids and proteins, which is a good reflection of lung function. Lung cancer tissue will also affect the biological components in sputum. The detection of bioactive substances in sputum can contribute to the diagnosis of lung cancer. Based on the current research results at home and abroad, this paper reviews the bioactive substances in sputum that can be used for the diagnosis of lung cancer.

## 1 背景

肺癌是目前全球死亡率最高的癌症^[1]^。当前大部分肺癌患者在诊断时已经处于晚期，晚期肺癌患者5年生存率不足15%，而Ia期肺癌患者的5年生存率可以超过80%^[2, 3]^。因此，如何尽早发现并诊断肺癌成为提高患者生存率的关键。

随着诊断技术的发展，早期肺癌筛查在过去的几十年内有了一定的发展与进步，胸部X射线、计算机断层扫描（computed tomography, CT）、低剂量螺旋CT（low-dose spiral CT, LDCT）、正电子发射断层扫描（positron emission tomography, PET）等技术的发展使得肺癌患者的生存率逐渐提高，但肺癌确诊的金标准仍为明确的病理结果，有创操作的风险依旧不能避免^[4, 5]^。细胞学检查能够为以上技术提供辅助诊断，但由于样本的获取及检测的局限，痰液细胞学的检测效力未能达到理想的效果^[6, 7]^。因此临床上迫切需要一种安全性更高、准确性更高的肺癌诊断方法。

随着对肺癌的分子机制研究的深入，基因组学技术的发展，更多生物分子被证实参与肿瘤进程，促进其发生发展。二代测序及深度检测技术的出现，大幅度提升了基因检测的敏感性和特异度^[8]^，而通过数据分析使得准确测定组织中肿瘤基因的拷贝数成为可能^[9]^。液体活检中基因相关样本检测的潜力凸显。利用患者的血液、胸腔积液、唾液、痰液等样本中提取的脱氧核糖核酸（deoxyribo nucleic acid, DNA）片段、微小RNA（microRNA, miRNA）、信使RNA（message RNA, mRNA）、蛋白质等能够获取肿瘤组织的遗传信息，该方法无创、易于获取、可重复多次提取，具有很强的应用前景^[10, 11]^。血液样本虽然易于提取，但其中相关基因产物含量低，且容易受机体其他代谢过程影响^[12]^；胸腔积液不易受其他过程影响，产物含量较高，但采集操作是有创性的^[13, 14]^。而痰液中产物含量较适宜，且无创不易受其他过程影响，含有多种生物活性物质，如蛋白质、DNA、miRNA、mRNA，是理想的液体活检样本。本文回顾了痰液中生物分子标记物在肺癌中的相关机制和应用价值。

## 2 痰液中的DNA

正常细胞在发生坏死和凋亡过程中可能会将部分DNA释放到内环境中，而肿瘤细胞释放的DNA片段带有肿瘤特有的遗传特征，对肿瘤的诊断具有重要价值。对含有肿瘤DNA的痰液进行检测，具有经济、安全、无创的特点，同时，肿瘤细胞遗传物质的改变通常发生于产生临床症状之前，对遗传物质的检测具有一定的前瞻预防性，而且，可多次反复取样还可以连续动态监测肿瘤基因，从而提示肿瘤的发生与发展^[7, 10, 15, 16]^。肿瘤细胞遗传物质DNA的改变是复杂且精密的，与癌症的发生发展密切相关，当前用于肺肿瘤细胞诊断的DNA遗传特征改变主要包括DNA的突变（DNA mutation）和异常甲基化（aberrant DNA methylation）。聚合酶链式反应（polymerase chain reaction, PCR）是检测DNA变异的常用技术，该技术简单易行、经济快速、敏感性较高^[17]^。DNA异常甲基化被认为是一种肿瘤细胞调控的机制，甲基化特异性PCR（methylation-specific PCR, MSP）是检测DNA甲基化的常用方法。

Keohavong等^[18, 19]^的工作证明了*KRAS*突变及*p53*突变可能适用于对肺癌高危人群中的痰液筛检，但当前对*KRAS*和*p53*基因检出时间和肺癌进展间关系的研究仍待完善。多项研究^[20-22]^显示，*p16*、*PAX5α*、*GATA5*、*SULF2*等基因的异常甲基化在肺癌早期筛查中的特异性超过70%。Hulbert等^[16]^的病例对照研究同样发现了*HOXA7*、*SOX17*等基因的异常甲基化检测的特异性超过80%。但这些基因用于筛查的敏感度不高，均低于70%。由于肺癌的发生涉及到复杂的遗传信息调控过程，当前的研究尝试将多种基因同时检测，以期提高敏感性，研究显示，同时对多个基因甲基化检测可以提高肺癌筛查和早期诊断的敏感性^[23, 24]^，可以将敏感性提高到98%，而机器学习的方法也可以通过多基因组合检测诊断出91%的肺癌^[16, 25, 26]^。痰液中多基因联合检测具有高特异性和敏感性，有望成为肺癌筛查和早期诊断的方向，需要更大样本、更多基因的联合研究和深入分析提供进一步的支持。

LDCT与痰液中DNA异常甲基化检测的结合也能使肺癌筛查的效率提高，对在LDCT上发现可疑结节的患者进行痰液生物活性物质检测能够得到63%-86%的敏感性和75%-92%的特异性^[16]^；Leng等^[27]^的研究也提示痰液生物活性物质检测能够排除1/3 LDCT的假阳性。而针对肺癌的诊断上，根据大量的病例研究和动物实验表明，原癌基因*KRAS*和抑癌基因*p53*在肺癌的发生发展中具有重要的作用^[28]^。Somers等^[29]^对肺腺癌患者的痰液进行了研究，结果提示*KRAS*基因的点突变可以在肺腺癌患者的痰液中检测到，为后续的检测的发展奠定了基础。Destro等^[18]^在一项对照实验中发现，肺癌患者中痰液样本检测出*KRAS*突变（79%）而对照组无一阳性，虽然当前针对痰液中KRAS和p53对于肺癌诊断的研究仍未有统一的结论，但KRAS和p53在肺癌发展中起着核心作用，结合痰液检测的无创性特点，痰液中KRAS和p53检测在今后肺癌的诊断技术中仍具有潜力。除了单基因检测，联合检测DNA甲基化和miRNA被证实可以显著提高检测的特异性和敏感性。研究^[30]^显示，同时检测2种miRNA（miR-31, miR-210）和2种DNA甲基化（*RASSF1A*和*3OST2*）能够获得87%的敏感度和90%的特异性。痰液中生物活性物质在肺癌诊断中具有很大的潜力，为了进一步验证检测的敏感度和特异性，需要多中心、大样本临床研究提供更充分的证据^[18, 19]^。

表皮生长因子受体（epidermal growth factor receptor, *EGFR*）相关基因突变被认为是肺腺癌发生的机制之一，痰液中*EGFR*突变对于诊断的影响也在研究之中，研究发现*EGFR*突变可能与女性或非吸烟患者的肺腺癌有关，肺癌的不同亚型可能会成为影响*EGFR*突变检出率的原因，同时，当前研究较小的样本量所能具备的代表性也值得进一步探究^[31-34]^。在临床治疗中，针对*EGFR*突变的靶向药被广泛证实有显著的疗效，*EGFR*突变的检测对非小细胞肺癌（non-small cell lung cancer, NSCLC）有着重要的作用^[35, 36]^，研究^[33]^显示，在已经明确诊断为*EGFR*突变型NSCLC的患者中，30%-50%的患者可以在痰液中检出这一突变，且所有对照组中均不能检出。另一项相似的研究的结果^[37]^显示，痰液中检测*EGFR*突变可以得到46.2%的敏感度和100%的特异性。虽然敏感度不够理想，但这些研究揭示了痰液*EGFR*突变检测可以在早期获得肺癌患者*EGFR*突变状态。间变性淋巴瘤激酶（anaplastic lymphoma kinase, *ALK*）也是NSCLC的常见治疗靶点，针对此基因突变的许多靶向疗法具有显著的疗效^[38]^，但当前尚未有针对痰液中*ALK*突变的检测研究。针对其他靶点如MET、BRAF的研究同样缺乏^[30]^。

## 3 痰液中的miRNA

miRNA是一种稳定的非编码单链小核苷酸序列，它们主要通过与位于靶mRNA 3’-非翻译区（untranslated area, UTR）内的种子序列结合来调控致癌和/或抑瘤基因，最终导致靶mRNA失活和降解，从而调节基因表达，参与调控肿瘤进程。大量研究^[39-41]^发现，miRNA基因的异常表达与肺癌的发生发展相关，一类miRNA作为抑癌基因发挥作用，如let-7家族（let-7a、let-7c）、miR-1^[42]^可抑制癌细胞的增殖及耐药，miRNA-1、miRNA-206^[43]^可通过减少肿瘤血管生成而抑制肿瘤的生长与转移，现发现miRNA-126、miRNA-494也有一定致癌作用^[44-47]^。另一类则作为致癌基因发挥作用，其高表达可促进肺癌的进展，如miR-103-a可增加血管生长因子的表达^[48]^，miR-31可引起肿瘤支持可溶性因子与侵袭性增加^[49]^，miRNA-17家族（miRNA-17、miRNA-18a、miRNA-19a、miRNA-20a、miRNA-19b-1、miRNA-92a-1）^[50]^、miRNA-335^[51]^、miRNA-155^[52]^、miRNA-183^[53]^均在肺癌组织中表达增加。

肺癌亚型传统上依赖于切除标本、支气管镜活检、细针穿刺的组织病理学观察，这些样本的采取方式对患者的侵袭性大^[54-56]^，然而研究人员^[57, 58]^发现，内源性miRNA以稳定的形式存在于痰液中，且对RNA酶具有抗性，此外在7 d内的痰液样本中也可以被检测出来，过去15年里痰液中miRNA检测越来越多地被用于肿瘤早期筛查与诊断。Zhang等^[59]^为评估痰液标本中miRNA的异常表达是否可以作为NSCLC的有用生物标志物，纳入了14篇文章，包括1, 009例NSCLC患者和1, 006例健康者对照，结果显示联合敏感性、特异性、阳性似然比（positive likelihood ratio, PLR）、阴性似然比（negative likelihood ratio, NLR）、诊断优势比（diagnostic odds ratio, DOR）和曲线下面积（area under the curve, AUC）分别为0.75（95%CI: 0.72-0.78）、0.88（95%CI: 0.86-0.90）、5.70（95%CI: 4.82-6.75）、0.30（95%CI: 0.26-0.34）、22.43（95%CI: 17.48-28.79）、0.89，提示痰液标本中的miRNA可能是NSCLC的无创性诊断生物标志物。

肺癌患者痰液中miRNA-21、miRNA-155、let-7a的含量与良性肺疾病患者与健康者的的痰液中含量差异显著，miRNA-21与miRNA-155在肺癌患者痰液中含量增高，而let-7a含量则相反^[60]^。Yu等^[58]^纳入36例肺癌患者和36例健康者对其痰液进行标记物优化，鉴定出7种表达显著改变的miRNA，4种在肺癌患者中高表达，3种低表达，最终选择4种miRNA（miRNA-21、miRNA-486、miRNA-200b和miRNA-375）组合，其对诊断肺腺癌的灵敏度和特异度分别为80.6%和91.7%。Roa等^[61]^检测肺癌患者痰液中5种miRNA（miR-21、miR-143、miR-155、miR-210、miR-372）含量增加，随后进行双盲定量分析，结果发现对诊断肺癌的敏感度和特异度分别为83.3%和100%。Xing等^[62]^通过实时定量反转录-聚合酶链反应（reverse transcription-polymerase chain reaction, RT-PCR）对48例Ⅰ期肺鳞癌患者和48例健康人的痰中miRNA标记，鉴定出6种miRNA，其中3种过度表达，另外3种表达不足，最终筛选出3种miRNA（miRNA-205、miRNA-210和miRNA-708），其在区分肺鳞癌患者和正常受试者方面具有73%的敏感性和96%的特异性。Ulivi等^[63]^和Su等^[64]^同样采用多种miRNA联合检测肺癌的方式，也拥有较好的检测能力。

提示痰液miRNA检测对肺癌的早期筛查与诊断有一定价值和意义，痰中的miRNA表达水平极为稳定，收集后1 d-7 d每天基本不会改变^[65]^。但该方法具有一定局限性，从患者身上采集的样本中细胞数量不同可能导致miRNA含量不稳定，且许多患者伴有呼吸肌疲劳、无力等症状，这使痰液的采集变得十分困难，部分患者痰液量减少甚至无痰。并且目前仅有少数miRNA能提供较好的诊断效果，而miRNA对癌症的调控作用也未能在诊断中有所反映。基于痰液miRNA对于肺癌诊断和筛查无疑是良好的工具，但对利用miRNA的定量分析对肺癌预后治疗、随访的评价作用未有报道，或许可成为研究的新方向。

## 4 痰液中的mRNA

mRNA，是由DNA单链作为模板通过转录产生、携带遗传信息、能参与蛋白质合成的一类单链核糖核酸。相较于miRNA，mRNA在痰液中降解快，因此有必要尽快对采集后的痰液标本进行检测或及时保存；目前常应用RT-PCR法对mRNA序列进行检测，对肺癌进行诊断和筛查。

Dong等^[67]^应用反转录PCR（reverse transcription-PCR, RT-PCR）法检测104例肺癌患者痰液标本，并与传统细胞学检测结果比较，肺癌患者痰标本Survivin mRNA的阳性率为60.6%（63/104），提示痰液mRNA作为肺癌筛查的可能性。Sun等^[68]^应用实时定量RT-PCR法检测了75例肺癌组织和71例相应痰标本中APRIL mRNA的表达，并进行相关性分析，结果表明肺癌、肺部良性疾病和健康志愿者的APRIL mRNA表达阳性率分别为81.7%（58/71）、3.2%（2/62）和1.5%（1/65）；肺癌患者痰液中APRIL mRNA的表达水平明显高于良性肺部疾病患者和健康志愿者。印记位点调节物兄弟因子（brother of the regulator of imprinted sites, BORIS）作为癌基因也在肺癌患者痰液中被检测到，BORIS阳性则有淋巴结转移、病理结果为进展期的提示^[69]^，提示了其在预后、治疗方面的作用。

Chen等^[70]^建立了一种新的PCR方法：Template-Ready PCR（TRPCR），提高了PCR的灵敏度；应用这种方法检测858例肺癌患者和480例非恶性肺部疾病志愿者的痰液标本中的人端粒酶逆转录酶（human telomerase reverse transcriptase, HTERT）mRNA，其阳性率分别为84.15%（722/858）和3.96%（19/480），差异有统计学意义。由于mRNA易降解的特性，其痰液检测受到限制，未有研究报道其生物学特性在肺癌复发、随访等方面的作用，但mRNA具有的基因特性仍具有较大潜力。

## 5 痰液中的蛋白质

21世纪以来，蛋白质组学技术不断得到发展与补充，利用该技术检测对应的生物标志物在疾病的筛查、诊断和预后中发挥着不可或缺的重要作用。肺癌患者的痰液中可以检测出敏感性好、特异性高的生物标志物，将会大大提高肺癌早期检测成功率，从而更好地进行肺癌的治疗和预后^[71]^。

Rangel等^[73]^利用透明质酸（hyaluronan, HA）区分肿瘤患者和无肿瘤患者的敏感性为80%，特异性为66%，拥有较好的敏感度，在肺癌筛查方面具有较大潜力。Yu等^[72]^研究了Ⅰ期肺癌患者的诊断方式，在痰上清液中利用蛋白质组学技术发现EN01蛋白水平增高，敏感性为58%，特异性为80%。Pio等^[74]^证实了肺癌患者痰中存在补体因子H的水平升高，补体因子H检验的敏感性和特异性分别为80%和88%；他们认为分子生物标记物的测量，如补体因子H，未来可能作为细胞学检测的辅助手段用于恶性肺部疾病的诊断。Li等^[75]^利用蛋白芯片和ELISA技术开发了三种肿瘤抗原相关自身抗体（tumor antigen-associated autoantibody, TAAb）DDX6、ENO1和14–3-3ζ作为诊断肺癌的生物标志物，其敏感性和特异性分别为81%和83%。

对痰液中蛋白质的检测拥有较高的特异性和敏感度，具有良好的诊断性作用，而通过蛋白质定量检测来判断肺癌进展程度的报道较少。

## 6 展望

痰液中生物标志物具有良好的肺癌诊断能力，但痰液标本收集则会干扰诊断的能力，故对痰液标本的收集是值得研究的方向。痰液中生物标志物大多为RNA，其稳定性不高，易分解，采样后及时检测也是提高检测能力的方法之一。血液中存在可评估肿瘤进展和治疗效果的生物标志物，而痰液中也可能存在类似的物质，除用于肺癌诊断也可用于对肺癌状态的实时检测。痰液中存在多种可用于检测的物质，联合诊断往往可以提高诊断的能力，目前较多研究为同类物质的联合检测，而不同物质如miRNA联合蛋白或miRNA联合DNA的检测方式还未有报道。同样生物活性物质的检测也可与LDCT联合诊断肺癌并提高二者的准确性^[16]^。

由于血清和血浆相比于痰和全血，具有更大的特异性和敏感性^[66]^，近年来对痰液生物活性物质的研究热度有所降低，较少研究利用定量检测对肺癌进程进行分析，而痰液中含有大量可检测的生物标志物，随着检测方式的不断改进，其在肺癌的诊断甚至治疗预后中的作用将会越来越大。
